# Social Engagement Among Family Caregivers: A Concept Analysis

**DOI:** 10.1002/nop2.70938

**Published:** 2026-11-13

**Authors:** Chih‐Yu Wang, Hyeyeon Shin, Kathy D. Wright

**Affiliations:** ^1^ College of Nursing The Ohio State University Columbus Ohio USA; ^2^ College of Medicine The Ohio State University Columbus Ohio USA

**Keywords:** caregivers, concept analysis, social engagement, social interaction, social support, stress

## Abstract

**Background:**

Social engagement among family caregivers is not well defined and is often used interchangeably with other terms.

**Aim:**

To clarify the concept of family caregivers' social engagement by identifying its defining attributes, antecedents, consequences, and empirical referents.

**Design:**

A concept analysis.

**Review Methods:**

This study followed Walker and Avant's method of concept analysis. Two authors independently screened titles and abstracts, followed by full texts, and extracted data for synthesis.

**Data Sources:**

Five databases, including PubMed, CINAHL, APA PsycINFO, Scopus, and Web of Science, were searched from inception through October 1, 2026.

**Results:**

Thirty‐one studies defined family caregivers' social engagement as active involvement with others in shared and supportive contexts, characterized by immediate meaningful experiences. Three attributes were identified: active involvement in social activities, shared and supportive contexts, and meaningful relationships. Four categories of antecedents were identified. Consequences included enhanced caregiver health and psychosocial well‐being, strengthened caregiving capacity, preserved identity, and improved care recipient health and well‐being. Empirical referents primarily assessed activity types, satisfaction or importance of activities, contact frequency, and social network size.

**Conclusion:**

This concept analysis clarifies family caregivers' social engagement and distinguishes it from related social concepts, including social interaction, social support, and social connectedness.

**Implications for the Profession and/or Patient Care:**

These findings may guide nurses in discussing caregivers' social needs, identifying barriers across dimensions, and supporting active engagement in meaningful activities that fit caregivers' circumstances.

**Impact:**

Social engagement among family caregivers remains conceptually ambiguous. This analysis identified three defining attributes and clarified its antecedents and consequences. The findings may inform nursing practice and future research to refine its conceptual boundaries with related social concepts and support future operationalization and empirical assessment.

**Reporting Method:**

We followed the Preferred Reporting Items for Systematic Reviews and Meta‐Analyses 2020 guidelines.

**No Patient or Public Contribution:**

This study was based exclusively on published literature; therefore, no patients, caregivers, or members of the public were involved in the research process.

## 1 Introduction

According to the report by the American Association of Retired Persons (AARP) and the National Alliance for Caregiving (2025), family caregivers are people who deliver unpaid assistance to their family members and friends who require supportive care. Their report estimates that 63 million adults are family caregivers providing care to adults and children, and that 61.1 million of these caregivers provide care without pay. The ongoing demands of family caregiving often limit caregivers' social and leisure time and constrain attention to their own needs, negatively influencing their psychosocial well‐being (Duplantier and Williamson 2023; Kotwal et al. 2024). Social engagement has therefore been proposed as a means to address these issues by fostering meaningful relationships (Liu et al. 2023).

Prior studies indicate that social engagement could buffer the adverse effects of caregiving stress on family caregivers' health. For example, dementia caregivers with higher levels of social engagement tend to report more positive caregiving experiences, contributing to reduced distress (Liu et al. 2023). Greater social engagement has also been shown to reduce caregiving burden, thereby improving sleep quality (Liang et al. 2020). Conversely, the Stress Process Model posits that the constriction of caregivers' social and recreational activities can lead to poorer health outcomes among caregivers (Pearlin et al. 1990).

Social engagement is often used interchangeably with terms such as social interaction, social support, and social participation, leading to unclear conceptual definitions (Monteiro et al. 2024). Terminological ambiguity may pose significant challenges for building clear operational definitions for precise measurement and for synthesizing findings (Liao et al. 2024; Yen et al. 2022). Although some frameworks position social engagement as a subset or deeper form of social participation, its definitional boundaries remain unclear, warranting a formal concept analysis (Levasseur et al. 2022). Despite evidence of its physical and mental health benefits in caregiver research, social engagement remains conceptually underdeveloped. Therefore, the aims of this concept analysis were: (1) to define the concept of social engagement among family caregivers; and (2) to identify its attributes, antecedents, consequences, and empirical referents and systematically organize the influences on family caregivers' social engagement.

## 2 Methods

### 2.1 Approach

For the concept analysis, we followed Walker and Avant's (2019) eight‐step method, which involves: (1) selecting the concept; (2) clarifying the aims of the analysis; (3) identifying all uses of the concept; (4) identifying its defining attributes; (5) constructing a model case; (6) developing borderline, related, and contrary cases; (7) specifying antecedents and consequences; and (8) describing empirical referents. The approach was designed to clarify inconsistently used concepts and help distinguish the target concept from overlapping constructs. It has been widely used in numerous studies (Hertanti et al. 2025), including those examining related social concepts (Dehi Aroogh and Mohammadi Shahboulaghi 2020).

### 2.2 Data Sources and Selection Criteria

In this study, we searched five databases, including PubMed, CINAHL, APA PsycINFO, Scopus, and Web of Science, using two main keywords: caregivers as the population term and social engagement as the concept term to identify relevant literature. The entire search strategy, including the search terms and Boolean operators applied in each database, is presented in Table S1. The initial search was conducted from May 25 to June 12, 2025. Findings from the initial analysis were previously reported as a brief conference abstract (Wang et al. 2025). The present analysis includes an updated literature search on October 1, 2026, and is reported as a full‐length manuscript. We used the same search strategy and eligibility criteria and incorporated a revised synthesis of the evidence.

The selection criteria included: (1) family caregivers who provide unpaid care; (2) qualitative, quantitative, theoretical, conceptual, review, or protocol articles; and (3) publications in peer‐reviewed journals written in English. The exclusion criteria were: (1) studies focusing on formal or professional caregivers; (2) articles that did not clearly define or discuss social engagement among family caregivers in the full text; and (3) conference abstracts or reports. No date limits were applied, and the search included literature through October 1, 2026.

### 2.3 Selection Process

All literature search results were imported into Covidence (Veritas Health Innovation, Melbourne, Australia, 2025). After duplicates were removed in Covidence, two reviewers (C.W. and H.S.) independently screened titles, abstracts, and full texts for eligibility according to predefined inclusion criteria. Differences in judgement at any stage were resolved through discussion and consensus between the two reviewers. The study selection process is outlined in a PRISMA (Preferred Reporting Items for Systematic Reviews and Meta‐Analyses) flow diagram (Page et al. 2021; Figure 1).

**FIGURE 1:**
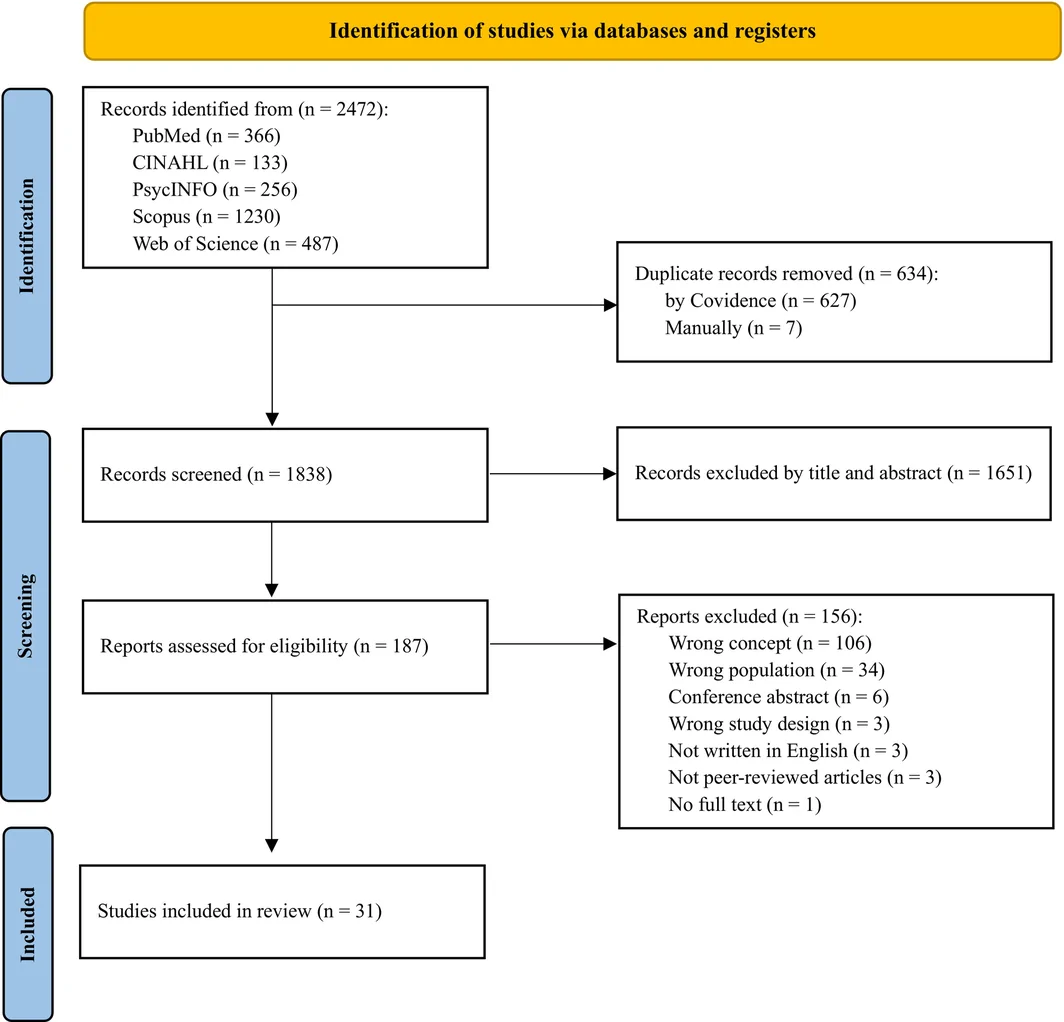
PRISMA flow diagram of the concept analysis study selection process.

### 2.4 Data Extraction and Synthesis

A total of 31 studies were identified for the final analysis. Two authors (C.W. and H.S.) independently examined and extracted content relevant to social engagement. In quantitative studies, we focused on how the concept was presented and elaborated in the introduction and discussion sections to capture its conceptual meaning. We also reviewed the methods section to identify any operational definitions or measures used to assess the concept. In qualitative studies, we examined not only how social engagement was framed in the introduction but also the related themes that emerged from interview data.

Detailed findings were recorded in an author‐developed Excel extraction template. The template included the following categories: title, authors, year, country, study design, research population, caregivers' age, critical components for conceptual analysis, antecedents, consequences, and empirical referents, along with illustrative quotations for each relevant domain. Attributes were derived from critical components, such as recurring conceptual and operational features of social engagement. Antecedents were circumstances or contextual factors that preceded the emergence of the concept. To guide the synthesis of antecedents, this study adopted the Stress Process Model (Pearlin et al. 1990) as an organizing framework. The model was selected because it is a caregiver‐specific framework that describes how stressors, contexts, and available resources interact within the caregiving experience. Within this framework, social engagement may function as a mediator between caregiving stressors and caregiver outcomes.

The identified antecedents were organized into four categories according to the four domains of the Stress Process Model: (1) background and context of stress, which include individual‐level characteristics, such as a person's sociodemographic factors, family structure, and baseline health, as well as contextual factors, such as program availability; (2) primary stressors, which are the direct demands of caregiving and the care recipient's condition that place immediate strain on the caregivers; (3) secondary stressors, which are indirect strains that arise from the caregiving role; and (4) mediators, which are variables that buffer the relationship between caregiving stressors and caregivers' outcomes.

Consequences were effects that tended to follow the occurrence of the concept and were synthesized inductively based on conceptual similarities. Regarding empirical referents, the reviewers recorded validated instruments or observable indicators used to assess caregivers' social engagement.

### 2.5 Validation Process

To enhance the rigour of the analysis and minimize potential interpretive bias, two authors (C.W. and H.S.) independently analysed the identified data and generated preliminary classifications. Several rounds of discussion were then held to resolve discrepancies until a consensus was reached. When necessary, the extracted information and original articles were re‐examined to ensure that the classifications remained grounded in the evidence. Through this iterative process, the data were synthesized into themes for attributes, categories for antecedents, domains of consequence, and a summary of empirical referents.

### 2.6 Ethical Considerations

This study was a concept analysis of previously published literature and involved no human participants or identifiable data. Therefore, ethical approval was not required.

## 3 Results

### 3.1 Study Characteristics

In total, 31 studies were included in the final concept analysis. Most were conducted in the United States (*n* = 15, 48.4%) and primarily used quantitative designs (*n* = 18, 58.1%). More than half of the studies focused on family caregivers of people living with dementia (*n* = 14, 45.2%). Additional studies examined caregivers of mixed populations (*n* = 6, 19.4%) and older adults (*n* = 3, 9.7%), and grandparents raising grandchildren (*n* = 4, 12.9%), while the remaining studies involved caregivers of cancer patients and haematopoietic stem cell transplantation (HSCT) survivors (Table 1).

**TABLE 1:** Characteristics and conceptual elements of included studies (*n* = 31).

Characteristic	*n*	(%)
Country		
United states	15	48.4
Canada	3	9.7
Australia	6	19.4
Europe	4	12.9
Asia	2	6.4
Africa	1	3.2
Study design		
Quantitative	18	58.1
Qualitative	8	25.8
Mixed‐methods	3	9.7
Systematic review	1	3.2
Concept analysis	1	3.2
Study population		
Caregivers of PLWD	14	45.2
Caregivers of mixed populations	6	19.4
Caregivers of older adults	3	9.7
Caregivers of cancer patients	3	9.7
Caregivers of HSCT survivors	1	3.2
Grandparents	4	12.9
Attributes		
Active involvement in social activities	26	83.9
Shared and supportive contexts	10	32.3
Meaningful relationship	15	48.4
Antecedents	16	51.6
Consequences	20	64.5
Empirical referents	17	54.8

Abbreviations: HSCT, haematopoietic stem cell transplantation; PLWD, people living with dementia.

Regarding the conceptual elements, all included studies reported at least one of the three attributes. Sixteen studies examined antecedents of social engagement among family caregivers, while 20 studies examined consequences grouped into four categories. In addition, 17 articles identified empirical referents for measuring this concept (Table 1).

### 3.2 All Uses of the Concept

Because social engagement among family caregivers has not been explicitly defined, we used Walker and Avant's (2019) method to examine how the concept has been applied across contexts and disciplines.

Among the studies included, more than half focused on caregivers of people living with dementia. Many studies also examined caregiving among older adults, either as care recipients or as caregivers, including grandparents (Table 1). Therefore, the use of social engagement in the caregiving literature is closely intertwined with aging‐ and dementia‐related contexts.

In aging research, social engagement has often been conflated with other social constructs, such as social networks, social support, and social interaction (Levasseur et al. 2022). It was sometimes treated as a higher level of social participation, emphasizing active involvement in social activities and meaningful relationships (Levasseur et al. 2010). On the other hand, for people living with dementia, social engagement was defined as emotional and behavioural responses elicited by meaningful social or activity‐based interactions that led to satisfaction (Matcham and Thoman 2022). These conceptualizations share similarities with those in the caregiving literature.

Among quantitative studies using secondary data, social engagement among family caregivers was commonly defined as engagement with social resources (Kelley et al. 2017; Liang et al. 2020; Litzelman et al. 2020). Constrained by the measures available in large‐scale datasets, social engagement was usually indexed by the number of activity types, frequency of contact, or social network size. For instance, datasets captured information on the activities participants routinely engaged in, including religious services, volunteer work, social clubs, and support groups (Kelley et al. 2017; Liang et al. 2020; Litzelman et al. 2020). Others recorded caregivers' self‐reported numbers of close friends and close relatives with whom they interacted during a specified time frame (Gorenko et al. 2024; Liu et al. 2021, 2023; Rozario and Simpson 2018). Social engagement was also described through caregiving‐related involvement with others. Prior studies showed that grand parenting can improve grandparents' cognitive health (Burn and Szoeke 2015; Jennings et al. 2021; Xu 2022) and well‐being (Notter 2022) through social activities like sharing important life values or casually connecting with neighbours while taking grandchildren for a walk (Xu 2022). However, it is worth noting that grandparents who did not live with their grandchildren or had less frequent contact often showed better health outcomes, suggesting that relationship quality might matter more than interaction quantity (Burn and Szoeke 2015; Xu 2022).

In contrast to quantitative studies that focus on the types, frequency, or size of social engagement, qualitative studies describe the settings and circumstances in which social engagement occurs. Cunningham et al. (2017) reported that the resilience‐building camp allowed young caregivers (aged 12 to 14 years) to step away from caregiving duties and engage socially with peers of similar age and caregiving experiences. The dance class for people living with dementia provided caregivers with opportunities to share their caregiving experiences and openly discuss the challenges of caring for their loved ones (Petts and Urmston 2022). Other qualitative findings illustrate family caregivers' lived and relational experiences of social engagement. At home, mealtimes offered a calm setting where caregiving dyads could nurture their mutual interactions while welcoming extended family members to share meals and strengthen broader family connections (Keller et al. 2015). The therapeutic choir for people living with dementia and their caregivers attracted active engagement from participants who enjoyed singing (Tamplin et al. 2018). Family caregivers described feelings of relatedness and enjoyment during singing and reported enhanced connection with their care recipients (Tamplin et al. 2024). In summary, the qualitative evidence demonstrates that caregiver social engagement is an experiential phenomenon tied to supportive atmospheres and shared activities.

Family caregiving research has also examined factors that influence social engagement and its implications for caregivers' physical and mental well‐being. Social engagement can be affected by the care recipient's poor health condition, distressing caregiving experiences, and internal conflicts between maintaining a sense of normalcy and seeking support (Litzelman et al. 2020). Long caregiving hours leave caregivers little time for other activities, and limited access to resources in non‐urban areas may constrain their willingness to engage in social activities (Liu et al. 2021). In contrast, higher levels of social engagement could buffer the effect of care recipient symptom severity on caregiver distress (Gorenko et al. 2024) and improve caregivers' quality of life (Mukuria et al. 2022). Increased social engagement was also positively associated with better health in both caregivers (Rozario and Simpson 2018) and care recipients (Amonoo et al. 2024). It helped maintain care recipients' cognitive status (Keller et al. 2015) while keeping caregivers cognitively active and stimulated (Xu 2022).

### 3.3 The Defining Attributes

The recurring features of attributes help define social engagement in family caregiving. In this analysis, we synthesized three attributes. Table S2 details each attribute with example quotations from the included studies. The attributes are described below.

#### 3.3.1 Active Involvement in Social Activities

Active involvement in social activities is the most frequently discussed attribute (*n* = 26) (Table 1). It refers to caregivers' observable actions as they actively engage with others in their social environment, which also reflects their access to available social resources (Babenko et al. 2026; Kelley et al. 2017; Liang et al. 2020; Litzelman et al. 2020; Wang et al. 2026; Zhong et al. 2026). These involvements can take many forms, such as informal, formal, and caregiving‐related activities. Informal activities may involve attending family events (Babenko et al. 2026; Laditka et al. 2012; Liang et al. 2020; Notter 2022; Rosenberg et al. 2026; Wang et al. 2026), meeting friends (Babenko et al. 2026; Gudenkauf et al. 2024; Laditka et al. 2012; Liang et al. 2020; Notter 2022), enjoying leisure exercises (Gudenkauf et al. 2024; Keller et al. 2015; Rosenberg et al. 2026; Schmidt et al. 2016), participating in clubs (Liang et al. 2020; Litzelman et al. 2020; Schmidt et al. 2016; Rosenberg et al. 2026), Bible study groups (Ali et al. 2023; Litzelman et al. 2020) or volunteer activities (Ali et al. 2023; Babenko et al. 2026; Notter 2022; Schmidt et al. 2016; Zhong et al. 2026). Formal activities were related to organized programs, such as formal volunteer programs (Laditka et al. 2012; Notter 2022; Schmidt et al. 2016), structured events at senior centers (Laditka et al. 2012; Liang et al. 2020), religious services (Babenko et al. 2026; Notter 2022; Rosenberg et al. 2026; Zhong et al. 2026), or intervention‐based activities such as community dances (Petts and Urmston 2022), therapeutic group singing (Tamplin et al. 2018, 2024), Dementia Cafés (community‐based social gatherings designed for people living with dementia and their caregivers), and resilience‐building camps (Cunningham et al. 2017). For caregiving‐related activities, these included task‐oriented care provided by family caregivers and meaningful engagement embedded in the caregiving experience, such as caring for grandchildren, that fostered relationships among caregivers, care recipients, and others (Burn and Szoeke 2015; Jennings et al. 2021; Xu 2022).

#### 3.3.2 Shared and Supportive Contexts

Shared and supportive contexts refer to familiar environments in which caregivers feel safe and supported when engaging with others (Amonoo et al. 2024; Cunningham et al. 2017; Keller et al. 2015; Liang et al. 2020; Masoud et al. 2021; van Roij et al. 2019), such as community spaces shared with others in similar caregiving roles (Cunningham et al. 2017; Masoud et al. 2021) or workplaces that allow them to experience normality and respite (van Roij et al. 2019). The online format of Dementia Cafés during the pandemic offered a judgement‐free space for caregiving dyads to connect and discuss challenges with peers (Masoud et al. 2021). For cancer caregivers, staying in the workforce provided opportunities to communicate with colleagues and maintain a balanced lifestyle beyond their caregiving role (Ali et al. 2023).

#### 3.3.3 Meaningful Relationships

Meaningful relationships are characterized by caregivers' interpersonal experiences and subjective perceptions during social engagement. This attribute reflects the perceived meaningfulness and quality of these engagements (Brookman et al. 2023; Keller et al. 2015; Liang et al. 2020; Liu et al. 2021; Masoud et al. 2021; Mukuria et al. 2022). Among the included studies, qualitative findings illustrated that caregivers often reported a sense of immediate mutual acceptance, emotional resonance, and shared enjoyment (Cunningham et al. 2017; Dai and Moffatt 2023; Tamplin et al. 2018) while actively engaging with others. Quantitative studies operationalized this subjective experience using measures of satisfaction with activities (Litzelman et al. 2020; Liu et al. 2021) and importance of activities (Rosenberg et al. 2026).

### 3.4 Model Case

According to Walker and Avant's (2019) concept analysis method, a model case is an exemplar that includes all defining attributes within a specific context to enhance understanding of the concept.

In our fictional model case, Mrs. A, a 62‐year‐old woman, provides care for her husband with dementia. Although caregiving occupies much of her daily life, she continues to attend a monthly caregiver support group. This group provides a safe space to talk about their personal struggles and learn from others with similar caregiving experiences. She also takes her husband to church on Sundays, where she finds joy in singing hymns with fellow church members and gathering together to pray for one another. Furthermore, she maintains contact with two close friends by meeting them for lunch. During these meals, they openly share their feelings and emotions, encourage one another, and provide mutual support. She believes that these meaningful relationships and mutual acceptance developed through social engagement help her cope with the demands of caregiving.

### 3.5 Additional Cases

Additional cases help distinguish the concept from similar or contrasting phenomena, including borderline, related, and contrary cases (Walker and Avant 2019).

#### 3.5.1 Borderline Case

A borderline case includes some, but not all, defining attributes and functions to illustrate the grey area of the concept (Walker and Avant 2019).

Mr. B is a 70‐year‐old man caring for his wife with early‐stage dementia. He usually accompanies her to medical appointments and brings her to dementia‐friendly activities at a community center. During these activities, Mr. B exchanges caregiving experiences with other caregivers and learns practical tips for daily care.

This case is considered borderline because it exhibits attributes of active involvement in social activities and of shared, supportive contexts. However, it lacks indications of meaningful relationships.

#### 3.5.2 Related Case

A related case is one that appears similar to the concept but lacks its key defining attributes (Walker and Avant 2019).

Mrs. C is a 45‐year‐old single mother caring for her mother with dementia. At the same time, she raises her two children and works part‐time. To manage her dual responsibilities, Mrs. C receives various supports from community resources, such as childcare, transportation to appointments, and respite care. This support helps her maintain a sense of normality by allowing her to continue working and to have some time for herself.

This case is viewed as a related case because it primarily reflects receiving social support from the social environment rather than actively engaging in social activities that characterize social engagement.

#### 3.5.3 Contrary Case

A contrary case is an example in which none of the defining attributes of the concept are present (Walker and Avant 2019).

Mr. D is a 76‐year‐old man caring for his wife with advanced dementia. He spends nearly all of his time at home and has withdrawn from social activities. Hence, he feels lonely and isolated.

### 3.6 Antecedents

Given the wide range of factors influencing social engagement identified in the literature, we adopted the Stress Process Model (Pearlin et al. 1990) to organize them into four categories of antecedents: background and context, primary stressors, secondary stressors, and mediators (Table 2). The identified factors in each study, with corresponding example quotations, are detailed in Table S2.

**TABLE 2:** Antecedents of social engagement among family caregivers.

Category	Antecedents
Background and context	Caregiver level: Age Gender Marital status Baseline health (e.g., comorbidities) Educational status Low technological literacy Employment status Income Care recipient level: Baseline health (e.g., comorbidities) Care recipient condition Dyadic level: Caregiving relationship Household size Geographical region Government financial support Technological infrastructure COVID‐19 pandemic
Primary stressors	Care recipient level: Cognitive status ADL Health condition (Overall health, BPSD)
Secondary stressors	Caregiver level: Constriction of social life Job‐caregiving conflict Intrapsychic strain Role transition
Mediators	Caregiver level: Coping strategy Dyadic level: Informal/Formal engagement Engagement activity group size Engagement activity components

*Note:* Antecedents were organized according to the domains of the Stress Process Model.

Abbreviations: ADL, activities of daily living; BPSD, behavioural and psychological symptoms of dementia.

#### 3.6.1 Background and Context

Within the background and context category, the antecedents at the caregiver level include their age (Litzelman et al. 2020; Liu et al. 2021), gender (Litzelman et al. 2020; Notter 2022; Schmidt et al. 2016; Xu 2022), marital status (Schmidt et al. 2016), baseline health (Litzelman et al. 2020; Schmidt et al. 2016), educational status (Schmidt et al. 2016), low technological literacy (Dai and Moffatt 2023), and employment status and income (Schmidt et al. 2016). At the care recipient level, the antecedents include their baseline health (Litzelman et al. 2020) and care recipient condition (Liu et al. 2021). At the dyadic level, the antecedents are the caregiving relationship (Liu et al. 2021) and household size (Schmidt et al. 2016). At the broader level, contextual antecedents include geographical region (Liu et al. 2021; Rozario and Simpson 2018), government financial support (Petts and Urmston 2022), technological infrastructure (Dai and Moffatt 2023; Masoud et al. 2021), and the COVID‐19 pandemic (Ali et al. 2023; Dai and Moffatt 2023).

#### 3.6.2 Primary Stressors

Primary stressors are the direct demands of caregiving arising from the care recipient's condition. These antecedents include the care recipient's cognitive status (Keller et al. 2015), ADL status (Keller et al. 2015), current health status (Amonoo et al. 2024), and behavioural symptoms (Masoud et al. 2021).

#### 3.6.3 Secondary Stressors

Secondary stressors reflect the indirect strains from the primary caregiving demands. These antecedents include caregivers' constriction of social life (Amonoo et al. 2024; Liang et al. 2020; Litzelman et al. 2020; Liu et al. 2021; Rosenberg et al. 2026; van Roij et al. 2019), intrapsychic strain (van Roij et al. 2019), job‐caregiving conflict (Amonoo et al. 2024), and role transition (Liu et al. 2021).

#### 3.6.4 Mediators

Mediating antecedents that buffer the relationship between caregiving stressors and outcomes include caregivers' coping strategies, the dyadic engagement of care recipients in informal or formal activities (Litzelman et al. 2020), and the group size (Tamplin et al. 2018) and components (Amonoo et al. 2024) of engagement activities.

### 3.7 Consequences

Based on the literature, the consequences of family caregivers' social engagement were grouped into four categories: enhanced caregiver health and psychosocial well‐being, strengthened caregiving capacity, preserved identity, and improved care recipient health and well‐being (Table 3). The identified consequences in each study, with corresponding example quotations, are detailed in Table S2.

**TABLE 3:** Consequences of social engagement among family caregivers.

Category	Consequences (*n*)
Enhanced caregiver health and psychosocial well‐being	Respite (4) Connectedness (3) Well‐being (2) Mental health (2) Quality of life (1) Reduced social isolation (1) Reduced distress (1) Reduced stress (2) Reduced anxiety (1) Reduced caregiving burden (2) Reduced depressive symptoms (1) Improved cognitive health (3) Improved general health (1)
Strengthened caregiving capacity	Foster interaction with professionals (1) Learning caregiving experience (1) Confidence (1)
Preserved identity	Caregiver identity (4) Care recipient identity (2)
Improved care recipient health and well‐being	Cognitive stimulation (2) Improved general health (1) Well‐being (1)

#### 3.7.1 Enhanced Caregiver Health and Psychosocial Well‐Being

Enhanced caregiver health and psychosocial well‐being refers to improvements in caregivers' physical, cognitive, and psychosocial outcomes associated with social engagement. The included studies reported that social engagement was associated with lower social isolation (Petts and Urmston 2022), distress (Gorenko et al. 2024), stress (Keller et al. 2015; Rosenberg et al. 2026), anxiety (Ali et al. 2023), depressive symptoms (Babenko et al. 2026), and caregiving burden (Rosenberg et al. 2026; Zhong et al. 2026). It was also described as providing opportunities for psychological respite by allowing caregivers to temporarily disengage from caregiving demands (Cunningham et al. 2017; Dai and Moffatt 2023; Masoud et al. 2021; Petts and Urmston 2022). Several studies of grandparent caregivers reported associations between grand parenting and better cognitive function (Burn and Szoeke 2015; Laditka et al. 2012; Xu 2022). Sustained engagement with peers experiencing similar caregiving challenges may help build social connectedness (Amonoo et al. 2024; Cunningham et al. 2017; Masoud et al. 2021). This enhanced sense of belonging and connection was further associated with quality of life and overall well‐being (Keller et al. 2015; Mukuria et al. 2022).

#### 3.7.2 Strengthened Caregiving Capacity

Caregiving capacity refers to caregivers' ability and confidence to manage caregiving responsibilities. During social engagement, caregivers reported acquiring practical knowledge and problem‐solving skills for managing caregiving challenges (Masoud et al. 2021). Sharing caregiving experiences and actively collaborating with healthcare professionals were linked to greater caregiver confidence (Dai and Moffatt 2023; Kelley et al. 2017).

#### 3.7.3 Preserved Identity

Social engagement may help preserve both caregivers' and care recipients' identities. Increased social engagement was associated with higher levels of positive aspects of caregiving and a stronger sense of self despite continuous caregiving stress (Keller et al. 2015; Liu et al. 2023; Petts and Urmston 2022). Conversely, reduced social engagement was linked to caregivers' loss of self, as their identity gradually becomes overshadowed by the caregiving role (Brookman et al. 2023). For care recipients, continued engagement in meaningful social activities may help preserve their identity despite disease progression (Keller et al. 2015; Petts and Urmston 2022).

#### 3.7.4 Improved Care Recipient Health and Well‐Being

Social engagement could also support care recipient health and well‐being. Active involvement in meaningful social activities involved cognitive stimulation and continued interaction with family members (Gorenko et al. 2024; Keller et al. 2015; Masoud et al. 2021). These experiences were associated with better overall health and well‐being (Dai and Moffatt 2023; Kelley et al. 2017).

### 3.8 Empirical Referents

Empirical referents were tools or instruments that reflected how the defining attributes of the concept can be measured in real‐world settings. Table 4 presents an overview of the empirical referents across the included studies. Seventeen of the 31 studies used empirical referents to operationalize at least one of the three proposed defining attributes of family caregivers' social engagement. Most studies relied on closed‐ended questions, with items ranging from one to eight. Response formats included numeric entry, binary (yes/no), and various point‐based Likert scales.

**TABLE 4:** Studies reporting empirical referents for family caregiver social engagement (*n* = 17).

Author, year	Items	Response format	Operationalized defining attributes (operational concept)		
			Active involvement in social activities (number of activity types/frequency of contact)	Shared and supportive contexts (activity involving shared contexts/social network size)	Meaningful relationships (satisfaction with/importance of activities)
Babenko et al. 2026	5	4–7 options	Yes (Frequency)	No	No
Burn and Szoeke 2015	2	Binary Numeric entry	Yes (Frequency)	No	No
Gorenko et al. 2024	6	3‐point scale	Yes (Frequency)	Yes (Activity context)	No
Gudenkauf et al. 2024	6 (Others)	5‐point scale	No	Yes (Activity context)	No
Jennings et al. 2021	2	7‐point scale Numeric entry	Yes (Frequency)	No	No
Kelley et al. 2017	5	Binary	Yes (Types)	Yes (Activity context)	No
Liang et al. 2020	5	Binary	Yes (Types)	Yes (Activity context)	No
Litzelman et al. 2020	7 (Types) 1 (Satisfaction)	Binary 3‐point scale	Yes (Types)	Yes (Activity context)	Yes (Satisfaction with activities)
Liu et al. 2021	2 (Social network size) 1 (Frequency) 5 (Satisfaction)	Numeric entry 6‐point scale 3‐point scale	Yes (Frequency)	Yes (Activity context) (Social network size)	Yes (Satisfaction with activities)
Liu et al. 2023	2 (Social network size) 1 (Frequency)	Numeric entry 6‐point scale	Yes (Frequency)	Yes (Activity context) (Social network size)	No
Notter 2022	4	Binary	Yes (Types)	Yes (Activity context)	No
Rosenberg et al. 2026	4 (Types) 4 (Importance)	Binary 3‐point scale	Yes (Types)	No	Yes (Importance of activities)
Rozario and Simpson 2018	2	6‐point scale	Yes (Frequency)	Yes (Activity context)	No
Schmidt et al. 2016	2	Binary	Yes (Types)	No	No
Wei et al. 2023	1	Numeric entry	No	Yes (Social network size)	No
Xu 2022	2 (Others)	Binary Numeric entry	No	No	No
Zhong et al. 2026	3 (Frequency) 3 (Types) 1 (Social network size)	7‐point scale 6‐point scale	Yes (Types) (Frequency)	Yes (Social network size)	No
17 Articles	1–8	Numeric entry Binary Point‐based scales	7 (Types) 8 (Frequency)	9 (Activity context) 4 (Social network size)	2 (Satisfaction with activities) 1 (Importance of activities)

For active involvement in social activities, seven studies operationalized this attribute using the number of activity types (Kelley et al. 2017; Liang et al. 2020; Litzelman et al. 2020; Notter 2022; Rosenberg et al. 2026; Schmidt et al. 2016; Zhong et al. 2026), while eight used frequency of contact (Babenko et al. 2026; Burn and Szoeke 2015; Gorenko et al. 2024; Jennings et al. 2021; Liu et al. 2021, 2023; Rozario and Simpson 2018; Zhong et al. 2026). A common item for the number of activity types was: ‘How many types of social activities did the caregiver participate in during the past month?’, with binary (yes/no) responses for each listed activity. In one study, activity types were instead derived from frequency items by summing the number of activities engaged in at least once per week (Zhong et al. 2026). Frequency of contact was typically measured over a defined timeframe, such as per month, using various response formats.

Shared and supportive contexts were captured in nine studies through social activities such as visiting family and friends (Gorenko et al. 2024; Gudenkauf et al. 2024; Liu et al. 2021, 2023; Notter 2022), attending religious services (Kelley et al. 2017; Liang et al. 2020; Rozario and Simpson 2018), and participating in support groups (Litzelman et al. 2020). Social network size was also used as a structural indicator in four studies (Liu et al. 2021, 2023; Wei et al. 2023; Zhong et al. 2026).

Regarding meaningful relationships, the included studies often conceptualized this attribute as perceived relationship quality within social activities. Two studies operationalized it using satisfaction with activities (Litzelman et al. 2020; Liu et al. 2021). Satisfaction was assessed using a 3‐point Likert scale (0 = not at all/low, 2 = a lot/very) to evaluate caregivers' satisfaction with the time they spent in five types of social activities over the past month. Additionally, Rosenberg et al. (2026) assessed the importance of social activities using a 3‐point response scale (very important, somewhat important, or not so important). Two studies used other empirical referents to reflect the extent of social engagement (Gudenkauf et al. 2024; Xu 2022). For instance, grandparenting level was assessed by asking, ‘How many weeks and hours per week did you spend caregiving?’ This item reflected both intensity and duration (Xu 2022). Table S3 details each empirical referent with example quotations from the included studies.

### 3.9 Proposed Definition of the Concept

Social engagement among family caregivers refers to their active involvement with others in shared and supportive contexts, characterized by immediate experiences of mutual acceptance and perceived meaningfulness (Figure 2).

**FIGURE 2:**
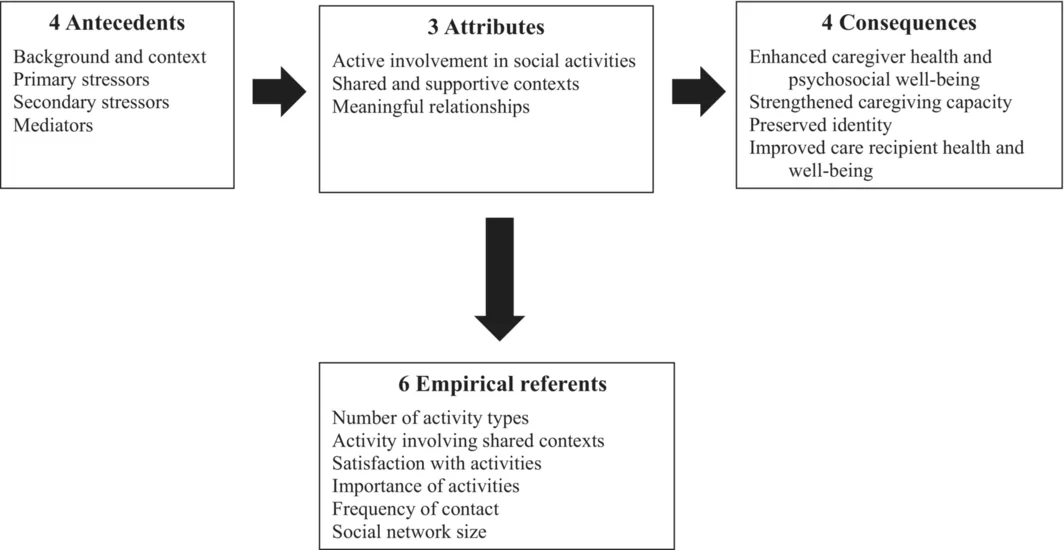
Conceptual model of social engagement among family caregivers.

## 4 Discussion

To the best of our knowledge, this concept analysis is the first to systematically examine the definition of social engagement among family caregivers. Since most studies examine only one or a few aspects of social engagement in the caregiving context, we used Walker and Avant's (2019) method to synthesize the evidence and propose a definition that offers a more comprehensive understanding of this concept.

Our findings indicate that active involvement in social activities is the most prominent attribute defining family caregivers' social engagement. Importantly, it reflects intentional behavioural investment within the relationship rather than the caregiver's mere physical presence (Burn and Szoeke 2015). For instance, one included study showed that caregivers actively engaged as content producers, listeners, and peer collaborators (Dai and Moffatt 2023). They openly shared personal stories and experiences during discussions and provided technical support to facilitators during virtual sessions. By emphasizing caregivers' active involvement in these social activities, this attribute clarified how social engagement differs from the passive receipt of social support (Levasseur et al. 2022). This distinction is also reflected in recent research that conceptualized and measured social engagement and social support as separate constructs (Babenko et al. 2026).

Regarding the forms of activities, the reviewed literature suggests that family caregivers' social engagement is not homogeneous. Although many studies report various types of activities, few explore how caregivers actively navigate among them in response to their personal contexts. Litzelman et al. (2020) found that lung cancer caregivers often preferred informal activities, such as religious practices, over formal support groups or counselling. Their choice of different forms of social engagement was associated with their health status and satisfaction with the caregiving help they received. For caregiving‐related activities, the included studies primarily focused on grandparent caregivers caring for their grandchildren (Burn and Szoeke 2015; Jennings et al. 2021; Xu 2022). This form of engagement was found to foster meaningful intergenerational relationships while providing emotional and cognitive benefits for grandparents. Nevertheless, Burn and Szoeke (2015) found that more frequent grandchild caregiving was associated with poorer cognitive function, suggesting that this form of engagement may also become a source of stress. When caregiving demands outweigh the benefits derived from social engagement, the activity may become overwhelming. Together, these findings suggest that social engagement is context‐dependent. Future research is needed to examine how family caregivers' social engagement is expressed across different caregiving populations, thereby further refining the concept.

Our analysis reveals that caregivers' willingness to engage actively is influenced by psychological safety within the environment, shared caregiving situations, and other contextual factors such as age and caregiving role. Caregivers tend to seek stigma‐free places with a similar caregiving identity rather than generic public spaces. For dementia caregivers, Memory Cafés create a safe space to discuss sensitive topics and exchange experiences, while also providing a temporary reprieve from caregiving stressors (Masoud et al. 2021). For young caregivers (aged 12 to 14 years), forming friendships is an important developmental task, yet caregiving responsibilities often disrupt these experiences (Cunningham et al. 2017). Engaging in a shared and supportive context provides a secure platform for them to confide in peers facing similar situations.

Meaningful relationships are a defining attribute of family caregivers' social engagement. Beyond social interaction, which refers to basic interpersonal exchanges through verbal and nonverbal communication (Hadley et al. 2022), social engagement emphasizes the interpersonal perceptions and the immediate emotional experiences embedded within those interactions. For instance, at the moment of engagement in resilience‐building camps, teenage caregivers (aged 12 to 14 years) derived enjoyment from feeling accepted and forming friendships with peers in similar situations (Cunningham et al. 2017). Similarly, in supportive therapeutic choirs, caregivers of people living with dementia found joy and connection while singing alongside their care recipients (Tamplin et al. 2018). Over time, these repeated experiences build stronger social connectedness, which represents the long‐term psychological outcome of engagement (Amonoo et al. 2024; Masoud et al. 2021; Petts and Urmston 2022; van Roij et al. 2019). Indeed, even when activities provide immediate enjoyment and acceptance, caregivers may need time to feel part of a group (Masoud et al. 2021).

Regarding the perceived quality of engagement, Liu et al. (2021) found that although caregivers' social network size declined over time, they maintained frequent contact with a small core group, which supports the importance of perceived meaningfulness and quality of social engagement. Quantitative studies operationalized this subjective dimension using satisfaction with activities and importance of activities. Notably, Rosenberg et al. (2026) found that perceived importance was associated with greater caregiver burden when religious activities were restricted, while importance did not differentiate burden for other types of social activities. This finding suggests that the relevance of perceived importance may vary by activity type. Further research should examine how meaningfulness is experienced in different types of activities and whether satisfaction and importance adequately capture the meaningfulness of social engagement among family caregivers.

Overall, shared and supportive contexts and meaningful relationships reflect the contextual and relational dimensions of social engagement. These attributes extend prior conceptualizations of social engagement, which had focused on the frequency of social contact (Gorenko et al. 2024; Jennings et al. 2021). Hence, identifying these attributes helps distinguish social engagement from social interaction and social connectedness. Collectively, the three defining attributes capture the core features of caregivers' social engagement and help differentiate it from related social terms that are often conflated in the literature.

Among the four categories of antecedents, background and context is the most frequently identified category. Social network size tends to become smaller with increasing age as older adults prioritize emotionally meaningful relationships. However, older caregivers may report greater satisfaction with social activities because they appraise their caregiving experiences more positively. In contrast, spousal caregivers reported lower satisfaction with social activities than other caregiver groups, possibly reflecting the greater caregiving burden typically associated with spousal caregiving (Liu et al. 2021). Some caregivers reported that limited access to devices or reliable internet constrained their engagement in virtual Dementia Cafés, while others found the online format more accessible for both caregivers and people living with dementia (Masoud et al. 2021). These findings demonstrate how sociodemographic characteristics and broader contextual factors can influence the social engagement of family caregivers.

The identified antecedent categories of primary and secondary stressors also reflect the sequential process described in the Stress Process Model (Pearlin et al. 1990), whereby primary stressors may give rise to secondary stressors, which subsequently shape caregivers' social engagement and, in turn, contribute to caregiver outcomes. Although progressive cognitive impairment (primary stressor) may limit social engagement among people living with dementia, caregivers often use adaptive strategies to accommodate these limitations (Gorenko et al. 2024). By choosing smaller social gatherings and quieter dining environments, caregivers were able to continue sharing meals with family and friends. These adaptations were associated with reduced caregiving stress, allowed care recipients to maintain their role as hosts, and supported dyadic personhood (Keller et al. 2015).

Another example is that worsening behavioural and psychological symptoms of dementia (primary stressor) may increase caregiving demands and responsibilities, leading to the constriction of caregivers' social lives (secondary stressor; Litzelman et al. 2020; Liu et al. 2021). Some caregivers reported that multiple caregiving tasks constrained their time, causing them to neglect their own physical needs, such as sleep, and therefore social engagement became a lower priority (Amonoo et al. 2024). This inability to balance multiple caregiving tasks and social roles has been recognized as a component of the broader construct of caregiver burden (Liang et al. 2020). Under this ongoing burden, caregivers may also feel guilty for engaging in activities rather than spending time with the care recipient (secondary stressor; van Roij et al. 2019). Even so, some caregivers sought opportunities for engagement because they perceived benefits for themselves and the care recipients (Dai and Moffatt 2023).

The mediator category of antecedents further suggests that characteristics of social activities may influence caregivers' social engagement. Caregivers reported that smaller group sizes facilitated stronger interpersonal bonds and helped avoid conflicts, reflecting the importance of meaningfulness in social engagement (Tamplin et al. 2018). Caregivers also emphasized that activities built on their prior experiences and everyday caregiving situations were more engaging (Amonoo et al. 2024). These tailored activity components may effectively foster caregivers' active involvement, which in turn strengthens their caregiving capacity and well‐being (Cunningham et al. 2017). Therefore, future research is needed to identify which aspects of social engagement best support meaningful engagement among family caregivers.

Beyond these antecedents, although cultural influences were not identified in the included studies, previous research suggests that cultural values and beliefs may influence how family caregivers engage in activities (Kim et al. 2026; Wright et al. 2025). Within African American communities, churches often serve as important centers of social, political, and economic support. Religious doctrines cultivate shared values, such as compassion and service to others, which may encourage caregivers to engage more deeply in church‐based social activities and foster close relationships (Kim et al. 2026). In some Asian cultures, concerns about shame and social judgement may cause caregivers to avoid social engagement, particularly when the care recipient displays socially unacceptable behaviours (Lwi et al. 2023). This chronic social withdrawal may lead to social isolation (Wei et al. 2023) and anxiety about how to return to a normal social life (Ali et al. 2023). The limited discussion of cultural influences may be due to previous studies examining related social concepts, such as social support and social participation. Future research is needed to explore the role of culture in shaping family caregivers' social engagement from a conceptual perspective.

Regarding empirical referents, none of the included studies used a validated instrument that comprehensively captured all three defining attributes of family caregivers' social engagement. Existing measures often relied on individual indicators that captured only specific aspects of the broader concept. However, frequency of contact reflects a behavioural aspect of social engagement and does not indicate active involvement (Gorenko et al. 2024; Jennings et al. 2021). Social network size represents the breadth of the social context but not shared experiences or supportive quality within it (Liu et al. 2023; Wei et al. 2023; Zhong et al. 2026). Meaningfulness was also less frequently assessed. Consequently, the present findings provide a conceptual foundation for future operationalization and empirical assessment of social engagement among family caregivers. Future studies are encouraged to further explore how the extent of social engagement varies across caregiving contexts.

### 4.1 Limitations

One limitation of this concept analysis was its restriction to English‐language articles, which may have influenced the generalizability of the findings to other cultural contexts. Another limitation was that we included only articles that used the term social engagement and discussed it in the full text. This criterion may have resulted in a relatively strict search strategy that excluded studies describing similar concepts using different terminology and may have influenced the identified attributes. Future research can build on this work by examining studies that capture this concept under related terms, which may help identify additional defining attributes, antecedents, and consequences.

### 4.2 Implications for Nursing Practice

The findings of this concept analysis may inform nursing practice by highlighting the multidimensional nature of family caregivers' social engagement. Nurses may use these dimensions to guide conversations with caregivers about their social experiences, needs, and preferences. These discussions can help identify barriers across different aspects of social engagement and inform individualized approaches. Nurses may then partner with caregivers to identify accessible activities and social resources, such as peer groups, community programs, and faith‐based organizations, that align with their interests, cultural context, and caregiving circumstances. Nurses may also encourage continued engagement in activities that caregivers perceive as meaningful to support sustained social connectedness over time. Finally, the three defining attributes identified in this analysis may provide an initial conceptual basis for future nursing assessment and instrument development while allowing for further refinement of the concept.

## 5 Conclusion

Social engagement among family caregivers refers to caregivers' active involvement with others in shared and supportive contexts, characterized by immediate experiences of mutual acceptance and perceived meaningfulness. This concept analysis clarified the defining attributes that distinguish family caregivers' social engagement from related concepts, such as the basic interpersonal exchanges characteristic of social interaction, the passive receipt of social support, and the long‐term sense of belonging characteristic of social connectedness. It also demonstrated how family caregivers' social engagement is shaped by diverse antecedents and contributes to important consequences for both caregivers and care recipients. Overall, these findings provide a clearer conceptual foundation for future research and practice.

## Author Contributions

**Chih‐Yu Wang:** conceptualization, methodology, data curation, formal analysis, writing – original draft, writing – review and editing, project administration. **Hyeyeon Shin:** conceptualization, methodology, formal analysis, data curation, writing – review and editing. **Kathy D. Wright:** conceptualization, methodology, data curation, writing – review and editing, supervision.

## Funding

The authors have nothing to report.

## Conflicts of Interest

The authors declare no conflicts of interest.

## Supporting information


**Table S1:** Search strategy used in five databases.
**Table S2:** Included articles for social engagement among family caregivers.
**Table S3:** Studies reporting empirical referents for family caregiver social engagement with corresponding example quotations.

## Data Availability

Data sharing not applicable to this article as no datasets were generated or analysed during the current study.
