# Supplementary material for: Social Engagement Among Family Caregivers: A Concept Analysis
Source: Nurs Open. 2026 Nov 13;13(11):e70938. doi: 10.1002/nop2.70938 (PMC13768589; doi:10.1002/nop2.70938)
Supplement: Supplementary file 1 [file NOP2-13-e70938-s001.docx]

**Table S1**

*Search strategy used in five databases*

| **Databases** | **Search** | **Query/Keywords** | **Results** |
| --- | --- | --- | --- |
| PubMed | #4 | Search updated through October 1, 2026 | 366 |
|  | #3 | Search: #1 AND #2 | 262 |
|  | #2 | Search: "Social engagement" [All Fields] | 2,993 |
|  | #1 | Search: ((((((caregivers[MeSH Terms]) OR (caregiver*)) OR (carer*)) OR (informal caregiver)) OR (spouse caregiver)) OR (unpaid)) OR (care partner*) | 247,578 |
| CINAHL | #4 | Search updated through October 1, 2026 | 133 |
|  | #3 | Search: #1 AND #2 NOT "social participation" | 117 |
|  | #2 | Search: "Social engagement" | 5,523 |
|  | #1 | Search: caregiver* OR carer* OR informal caregiver OR spouse caregiver OR unpaid OR care partner* | 141,851 |
| APA PsycINFO | #4 | Search updated through October 1, 2026 | 256 |
|  | #3 | Search: #1 AND #2 NOT "social participation" | 211 |
|  | #2 | Search: "Social engagement" | 3,261 |
|  | #1 | Search: caregiver* OR carer* OR informal caregiver OR spouse caregiver OR unpaid OR care partner* | 122,392 |
| Scopus | #4 | Search updated through October 1, 2026 | 1,230 |
|  | #3 | Search: #1 AND #2 AND NOT ALL "social participation" | 966 |
|  | #2 | Search: ALL ( "Social engagement" ) | 31,508 |
|  | #1 | Search: ALL (caregiver* OR carer* OR informal AND caregiver OR spouse AND caregiver OR unpaid OR care AND partner*) | 77,862 |
| Web of Science | #4 | Search updated through October 1, 2026 | 487 |
|  | #3 | Search: #1 AND #2 NOT "social participation" | 365 |
|  | #2 | Search: "Social engagement" | 6,615 |
|  | #1 | Search: caregiver* OR carer* OR informal caregiver OR spouse caregiver OR unpaid OR care partner* | 297,034 |

**Table S2**

*Included articles for social engagement among family caregivers*

| **No** | **Title (Authors, year)** | **Country,**  **study design,**  **population** | **Main elements for defining attributes** | **Conceptual elements**  **"Examples of quotes"** |
| --- | --- | --- | --- | --- |
| 1 | Navigating the COVID-19 pandemic together: Discussions between persons with early-stage dementia and their adult children (Ali et al., 2023) | United States,  Qualitative study,  Adult-child caregivers (mean age = 48.2 years) of PWD at an early stage | - Active involvement in social activities   Caregivers in this study mentioned that the COVID-19 pandemic reduced their opportunities for social engagement in both their personal and professional lives, as they were concerned about exposing the care recipient to the virus. Family caregivers were unable to engage in everyday activities such as eating at restaurants, attending Bible study groups, or participating in social and volunteer activities, and they could not meet with other family members in person. Additionally, the pandemic forced them to work remotely, leaving them with little personal time and no in-person interactions with colleagues, and they struggled to find childcare. | Antecedents:   - COVID-19 pandemic   *"Adult children shared with their parents how the pandemic resulted in reduced social engagement and challenging work arrangements."*  Consequences:   - Caregiver health and psychosocial well-being (increased anxiety associated with reduced social engagement)   *"The prolonged reduction in social interactions—initiated by participants themselves or*  *enforced by protocols levied by the government, workplace, or other businesses—was*  *a source of anxiety."* |
| 2 | A positive psychology intervention for caregivers of hematopoietic stem cell transplantation survivors (PATH-C): Initial testing and single-arm pilot trial (Amonoo et al., 2024) | United States,  Mixed-methods study,  Informal caregivers (mean age = 63.0 years) of hematopoietic stem cell transplantation (HSCT) survivors | - Shared and supportive contexts - Meaningful relationships   The qualitative part generated subthemes of social engagement from participants after they received the Positive Affect in the Transplantation of Hematopoietic Stem Cells (PATH) intervention via phone calls. Participants described how gratitude exercises in the intervention helped them engage with others who shared similar experiences, through which they developed a sense of relatedness, found meaning in life, and recognized the importance of social relationships. | Antecedents:   - Care recipient health condition   *"Several caregivers noted that the good health and uncomplicated post-transplant recovery of their loved one offered them more time to engage with PATH."*   - Constriction of social life   *"Given the multiple responsibilities that caregivers were balancing, in addition to their own physical needs like sleeping, engaging with an intervention that focused on psychological needs felt low priority to some caregivers."*   - Engagement activity components   *"I thought the three weeks in each [module] made sense. And I do think to say how can you apply this in your daily life was important too."*   - Job-caregiving conflict   *"My work schedule was changing every week because of where [my loved one] was. So there were times I just sat in my car when I had to do this because I had to step out of work."*   - Caregivers’ income   *"I don’t have any real pressing economic needs. It gives me the freedom to say, Well, what do I want to do with my time? How do I want to spend it?"*  Consequences:   - Caregiver health and psychosocial well-being (Connectedness)   *"Cultivating these relationships helped caregivers to feel more connected to the world around them."* |
| 3 | The role of social engagement and support in the association between combined caregiver–cancer survivor status and depressive symptoms  (Babenko et al., 2026) | United States,  Quantitative study,  Cancer survivor-caregivers (mean age = 73.0 years) of mixed populations | - Shared and supportive contexts   This secondary data analysis examined depressive symptoms among cancer survivors with caregiving responsibilities compared with three groups and explored whether social support and social engagement mediated these associations. Social engagement was conceptualized as a multidimensional construct reflecting active participation in social activities and relationships. It was operationalized using five indicators: religious service attendance, volunteering, participation in organized group meetings, socializing with friends or relatives, and visiting neighbors. Cancer survivor-caregivers reported higher depressive symptoms than all comparison groups. However, social engagement did not significantly mediate this association. Volunteering, socializing with friends or relatives, visiting neighbors, and attending organized group meetings were individually associated with fewer depressive symptoms. | Consequences:   - Caregiver health and psychosocial well-being (Depressive symptoms)   *"Higher levels of all social support and social engagement constructs (except for religious attendance) were associated with lower depressive symptoms."* |
| 4 | Technology for dementia care: What would good technology look like and do, from carers' perspectives? (Brookman et al., 2023) | Australia,  Mixed-methods study,  Informal caregivers of PWD (mean age = 60.0 years) | - Meaningful relationships   This study explored how assistive technology can support caregivers’ social engagement. In this context, caregivers emphasized that technology should primarily facilitate meaningful remote communication with family members, rather than simply assisting with daily or physical caregiving tasks, as a way to address social isolation and the strained relationships caused by caregiving responsibilities. Additionally, they suggested multimodal technologies that incorporate visual, auditory, or tactile features, and they preferred interaction with real people rather than artificial agents such as social robots, ideally without requiring staff involvement. | Consequences:   - Preserved identity **(**Caregiver)   *"I had gone from being an independent, active social person to just being a carer. Your will just goes down until your life is just this little pinprick. And you just have to dedicate yourself entirely to them."*  Empirical referents |
| 5 | Grandparenting predicts late-life cognition: Results from the Women's Healthy Ageing Project (Burn & Szoeke, 2015) | Australia,  Quantitative study,  Women caregivers (mean age = 60.0 years) of grandchildren | - Active involvement in social activities - Meaningful relationships   This study viewed grandparenting as a form of social engagement for grandparents. It specifically investigated the benefits of grandparenting for grandmothers over the age of 65 due to inconsistent findings on its impact on cognitive function. Social engagement was measured by asking participants whether they provided care for their grandchildren and how frequently they did so. The findings showed that participants who engaged in grandparenting had better episodic memory and cognitive function than those who did not. However, those with lower grandparenting frequency showed better cognitive outcomes than those with higher frequency, suggesting that the active relationship is more important than the quantity of contacts. | Consequences:   - Caregiver health and psychosocial well-being (Cognitive health)   *"Participants who were minding their grandchildren demonstrated the highest performance on both episodic memory and executive function tests."*  Empirical referents |
| 6 | A qualitative evaluation of an innovative resilience-building camp for young carers (Cunningham et al., 2017) | Australia,  Qualitative study,  Young carers (aged 12-14 years) who take care of parents, siblings or grandparents with chronic illness or disability | - Active involvement in social activities - Shared and supportive contexts - Meaningful relationships   In this study, the resilience-building camp offered young carers a short break from their caregiving responsibilities. It also provided opportunities for social engagement by enabling them to form friendships with peers of similar age who shared similar caregiving challenges. The program was described as both fun and valuable, as it provided a safe space for participants to share their unique challenges while building supportive connections. | Consequences:   - Caregiver health and psychosocial well-being **(**Respite)   *"It just helps you get your mind off a lot of stuff that’s going on."*   - Caregiver health and psychosocial well-being (Connectedness)   *"It not only allowed them uninterrupted time to build relationships but also allowed them to connect with others facing similar challenges."* |
| 7 | Enriching social sharing for the dementia community: Insights from in-person and online social programs (Dai & Moffatt, 2023) | Canada,  Qualitative study,  Informal caregivers (mean age = 65.0 years) of PWD at the early and middle stages | - Active involvement in social activities - Shared and supportive contexts   The Tales & Travels program provided social engagement opportunities for people with dementia and their caregivers, and this study compared the effectiveness of its in-person and virtual formats during the COVID-19 pandemic. The in-person format, a storytelling-oriented program, had more tangible materials and shared physical environments that allowed richer sensory experiences and promoted collaboration. In contrast, the online version was preferred only when it included physically engaging activities, such as virtual escape rooms and yoga, as it provided a refreshing break from caregiving routines. Additionally, caregivers frequently had to assist with technology use, which reshaped the dyadic relationship and fostered new forms of interaction with professionals. | Antecedents:   - Low technological literacy   *"[Some] have a tablet at home, but honestly, they just don’t know how to turn it on."*   - Access to formal activities (Technological infrastructure)   *"And focusing on the screen is already a lot of our clients don’t even have Internet at home."*   - Covid-19 pandemic   *"The dementia community faces major challenges in social engagements, which have been further complicated by the prolonged physical distancing measures due to the COVID-19 pandemic."*  Consequences:   - Care recipient health and well-being   *"Dyads described how it was beneficial and enjoyable for participants with dementia to remain socially active despite the burden it placed on their caregivers to manage these activities."*   - Caregiver health and psychosocial well-being (Respite)   *"It’s not a respite anymore for the caregiver but . . . a respite in the sense that they could derive something from it as well. . . they’re interacting with. . . their loved one or the person they’re caring for in a different way."*   - Strengthened caregiving capacity (Partnership with professionals)   *"Caregivers’ extended collaborator roles in the new virtual contexts of social experiences. Caregivers collaborate more with professionals."* |
| 8 | Social and leisure engagement moderates the association between care partner distress and cognitive status of care recipients with dementia (Gorenko et al., 2024) | Canada,  Quantitative study,  Informal caregivers (mean age = 67.4 years) of PWD | - Active involvement in social activities - Shared and supportive contexts   This longitudinal study investigated whether social engagement moderates the relationship between cognitive impairment in people with dementia and caregiver distress. Social engagement was described as social contact and participation in various leisure activities. A six-item scale measured the frequency of social activities over the past six months using a three-point scale, with higher scores indicating greater engagement. The research findings showed that PWDs’ cognitive function was negatively associated with caregivers’ distress between dyads, but no such association was observed within dyads over time. Furthermore, this relationship was moderated by social engagement, with greater engagement buffering the effect of cognitive impairment on caregiver distress. | Consequences:   - Care recipient health and well-being (Cognitive stimulation)   *"That is, social/leisure engagement for PWD may help modulate the deleterious impact of their cognitive impairment."*   - Caregiver health and psychosocial well-being (Distress)   *"Social/leisure engagement in both CP and PwD significantly moderated the influence of PwD cognitive status on CP distress between-dyads."*  Empirical referents |
| 9 | Feasibility and acceptability of C-PRIME: A health promotion intervention for family caregivers of patients with colorectal cancer (Gudenkauf et al., 2024) | United States,  Quantitative study,  Caregivers (mean age = 52.0 years) of patients with colorectal cancer | - Active involvement in social activities - Shared and supportive contexts   Social engagement was measured as one of the outcomes to evaluate the impact of a pilot digital health promotion intervention (C-PRIME) for caregivers. It was conceptualized as caregivers’ ability to participate in their regular leisure and social activities, such as spending time with friends or engaging in family events, while managing caregiving responsibilities. The results showed that, after the intervention, 23% of caregivers demonstrated improvement in their social engagement, supported by wearable behavior monitoring and weekly health coaching. | Empirical referents |
| 10 | Grandchild Caregiving and Cognitive Health Among Grandparents in Rural South Africa. (Jennings et al., 2021) | South Africa,  Quantitative study,  Caregivers (mean age = 63.8 years) of grandchildren | - Active involvement in social activities   This study proposed that the association between grandchild caregiving and grandparents' cognitive function would be mediated by social engagement, defined as social interaction and social support. Social interaction refers to opportunities for grandparents to engage with others when bringing grandchildren outside the home, while social support refers to receiving caregiving-related assistance from others. However, the results did not support this mediation hypothesis. | Empirical referents |
| 11 | Strategies to support engagement and continuity of activity during mealtimes for families living with dementia; a qualitative study (Keller et al., 2015) | Canada,  Qualitative study,  Informal caregivers (aged 30-88 years) of PWD | - Active involvement in social activities - Shared and supportive contexts - Meaningful relationships   Social engagement during mealtimes creates a safe and calm atmosphere for PWD and their caregivers to maintain continuous interaction. It fosters dignity and relationships through mutual communication, emotional support, and physical assistance during meals. In addition, mealtimes offer opportunities to connect with extended family members and social groups by eating out together and sharing caregiving challenges, thus reinforcing meaningful roles. | Antecedents:   - Care recipients’ ADL   *"Changes in eating conduct begin to occur while living in the community, potentially leading to the person with dementia eating meals in solitude because they or others are embarrassed or worried about eating at the family table or in public."*   - Care recipients’ cognitive status   *"As her dementia progressed, family gradually managed her changing capacity with respect to entertaining and chose to go to a club or restaurant for meals with her husband’s work associates."*  Consequences:   - Caregiver health and psychosocial well-being (Stress)   *"Go on special eating outings to alleviate daily stress."*   - Preserved identity (Care recipients)   *“Taking family out for a meal was a way for the person with dementia to feel like they were still hosting.”*   - Preserved identity (Caregivers)   *"We contend that these theoretically based strategies that are consistently and purposefully implemented during mealtimes will serve to honor identity."*   - Caregiver health and psychosocial well-being   *"Mealtimes provided a natural, normative*  *and guaranteed time for dyad participants to connect with each other and friends and family."*   - Caregiver health and psychosocial well-being   *"Completion of these activities provided a sense of accomplishment for both care partners and persons with dementia and also a point for connection, if done together."* |
| 12 | Perceived support from a caregiver's social ties predicts subsequent care-recipient health (Kelley et al., 2017) | United States,  Quantitative study,  Caregivers (mean age = 56.0 years) of older adults | - Active involvement in social activities - Shared and supportive contexts   This longitudinal study used secondary data to explore how various social resources influence care-recipient health. Social engagement is conceptualized as one of the resource domains within a caregiver's social support network. It is operationalized as one of the four social support domains and measured by caregivers' reported participation in five types of social activities in the past month. Findings showed that social engagement was positively associated with caregivers' confidence and directly predicted better care-recipient health at the time of the first interview. Moreover, social engagement continued to predict better health outcomes for care recipients one year later, after controlling for their initial health status. | Consequences:   - Strengthened caregiving capacity (Confidence)   *"Social engagement with members from caregivers' social support networks was positively associated with caregiver conﬁdence."*   - Care recipient health and well-being   *"Social engagement positively predicted patient health at time 2 controlling for time 1."*  Empirical referents |
| 13 | Beliefs about promoting cognitive health among Filipino Americans who care for persons with dementia (Laditka et al., 2012) | United States,  Qualitative study,  Filipino American caregivers (mean age = 58.0 years) of PWD | - Active involvement in social activities   In this study, social engagement was coded as one of the themes that support the promotion of cognitive health among Filipino American caregivers. It was described as social involvement and interaction, such as participating in activities at senior centers, making new friends, gathering with family members, and volunteering, all of which were perceived to benefit their cognitive functioning. | Consequences:   - Caregiver health and psychosocial well-being (Cognitive health)   *"Participants in all focus groups spoke about their beliefs about the benefits of social interaction for cognitive health."* |
| 14 | Association between role overload and sleep disturbance among dementia caregivers: The impact of social support and social engagement. (Liang et al., 2020) | United States,  Quantitative study,  Informal caregivers (mean age = 60.4 years) of PWD | - Active involvement in social activities - Shared and supportive contexts - Meaningful relationships   This study used secondary dyadic data to explore the relationship between role overload and sleep disturbance, and whether social support and social engagement moderate this relationship. Social engagement and social support are both categorized as forms of access to social resources. However, they differ in that social support refers to receiving assistance from one’s social network, whereas social engagement emphasizes active participation in meaningful activities to improve physical and psychosocial health. Social engagement includes informal activities, such as spending time with friends and family, and formal activities, such as attending organized programs or events. Results showed that social engagement did not moderate the relationship between role overload and sleep disturbance. Only instrumental support, within the social support domains, showed a moderating effect among adult child caregivers. | Antecedents:   - Constriction of social life   *"The analysis found a negative correlation between SE and RO. This implies that overloaded caregivers may have less time for social participation and experience tension in their social roles."*  Empirical referents |
| 15 | Trajectories of social resource use among informal lung cancer caregivers (Litzelman  et al., 2020) | United States,  Quantitative study,  Caregivers (mean age = 55.0 years) of patients with non–small cell lung cancer | - Active involvement in social activities - Shared and supportive contexts - Meaningful relationships   This longitudinal study investigated social resource use across the caregiving trajectory among lung cancer caregivers. Social engagement was conceptualized as engagement with social resources, including formal support from healthcare professionals and informal support from family and friends. Social engagement can be influenced by the care recipient’s poor health condition, distressing experiences, the internal conflict between maintaining a sense of normalcy and seeking support, as well as the caregiver’s satisfaction with the activities themselves and whether they meet the caregivers’ changing needs. Research findings showed that all caregivers engaged in at least one type of social resource, and their engagement increased over time. However, their level of engagement was much lower than that of the non-caregiver population. Additionally, caregivers tended to prefer informal social engagement over formal social engagement. | Antecedents:   - Care recipients’ baseline health (e.g. comorbidities)   *"Patient comorbidities were associated with a lower likelihood of caregivers’ formal resource use."*   - Caregivers’ baseline health   *"Caregivers with 1 or more health conditions were twice as likely to use formal resources as those with no health conditions."*   - Caregivers’ age   *"Caregivers who were older and female were more likely to report using informal resources."*   - Caregivers’ gender   *"Several predisposing and enabling/hindering factors emerged as key predictors of social resource use, including caregiver age and sex."*   - Care recipients’ informal/formal engagement   *"The patients’ use of social resources was a strong independent predictor of caregivers’ social resource use."*   - Constriction of social life   *"Myriad contextual factors likely play a role in caregivers’ choices around social activities, including time pressure and perceived strain."*   - Caregivers’ coping strategy   *"Increased social resource use may represent a return to caregivers’ usual patterns and routines as they adapt to the cancer diagnosis and the caregiving role."*  Empirical referents |
| 16 | Change in social engagement among incident caregivers and controls: Findings from the caregiving transitions study (Liu et al., 2021) | United States,  Quantitative study,  Caregivers (mean age = 71.8 years) of PWD | - Active involvement in social activities - Shared and supportive contexts - Meaningful relationships   This longitudinal study used secondary data to explore changes in social engagement among incident caregivers over time and compared them with matched non-caregiving controls. Social engagement was defined as both the level of involvement and the quality of engagement in social activities. In this study, social engagement was operationalized using three indicators: the size of the social network, the frequency of social contacts, and satisfaction with the level of social engagement. Research findings revealed that incident caregivers experienced significant declines over time in social network size and frequency of social contact, as well as lower satisfaction with social activities. Additionally, caregivers of people with dementia and those providing more caregiving hours per week showed greater reductions in social network size. Spousal caregivers, younger caregivers, those reporting higher caregiving strain, those with longer weekly caregiving hours, and those living in non-urban areas reported lower satisfaction with social activities. | Antecedents:   - Care recipient condition   *"Dementia caregivers actually reported less decline in social network size compared to nondementia caregivers."*   - Caregivers’ age   *"Older age was associated with higher satisfaction with social activities among incident caregivers."*   - Caregiving relationship   *"Spouse caregivers experience lower satisfaction with social activities than caregivers who were neither spouses nor adult children."*   - Constriction of social life   *"Incident caregivers likely had less time to participate in social activities due to caregiving responsibilities."*   - Geographical region   *"We found that caregivers residing in mixed areas, deﬁned as areas that are 25%–75% urban, reported signiﬁcantly lower satisfaction with social activities than caregivers in urban areas."*   - Role transition   *"Taking on responsibilities to provide ongoing care for a family member or friend with a disability is another transition that is associated with aging and can affect one’s engagement in social activities."*  Empirical referents |
| 17 | Positive aspects of caregiving in incident and long-term caregivers: Role of social engagement and distress (Liu et al., 2023) | United States,  Quantitative study,  Caregivers (mean age = 72.0 years) of PWD | - Active involvement in social activities - Shared and supportive contexts   This cross-sectional study used secondary data to examine how positive aspects of caregiving differ based on caregiving duration and levels of social engagement, while accounting for caregiver distress. Findings indicated that both social network size and frequency of social contact were positively associated with PAC after controlling for relevant covariates. | Consequences:   - Preserved identity (Caregiver)   *"The level of social engagement was strongly associated with positive aspects of caregiving even after accounting for levels of distress."*  Empirical referents |
| 18 | "We don't feel so alone": A qualitative study of virtual memory cafés to support social connectedness among individuals living with dementia and care partners during COVID-19 (Masoud et al., 2021) | United States,  Qualitative study,  Informal caregivers (spouses/adult children) of PWD | - Active involvement in social activities - Shared and supportive contexts - Meaningful relationships   This qualitative study describes how the online format of the Memory Café Network influenced social connectedness among caregiving dyads of persons living with dementia (PWD) during the COVID-19 pandemic. These Alzheimer’s or dementia cafés provided a safe and supportive environment for caregiving dyads to engage socially without fear or stigma, encouraging meaningful interaction with family members and other caregivers. | Antecedents:   - Care recipients’ behavioral symptoms   *"Several participants expressed that the virtual format is not accessible to their family member living with dementia due to behavioral symptoms or that they have a general disinterest in using the technology."*   - Access to formal activities   *"We’ve enjoyed the virtual Memory Café as is. I do know that mom and dad—it is more likely that they will attend via the Zoom than if it was in person. . . because to get mom out of the house, it becomes an ordeal."*   - Access to formal activities (Technological infrastructure)   *"Many simply do not have access to reliable internet or computers."*  Consequences:   - Caregiver health and psychosocial well-being (Respite)   *"The majority of interviewees expressed that Memory Cafés oﬀer some sense of reprieve from their typical day-to-day experiences."*   - Care recipient health and well-being (Cognitive stimulation)   *"We’re always looking for ways to keep active and keep social, so in that way, that’s one of the rules of being a caregiver is to try and ﬁnd those experiences and keep the brain working and stimulated."*   - Strengthened caregiving capacity (Learning caregiving experience)   *"That has helped because I understand more in seeing other people, how they deal with their, the person who has it. It gives me that experience."*   - Caregiver health and psychosocial well-being (Connectedness)   *"So, we are really glad that we found this Bilingual program and we appreciate that they will translate for us because she feels more included."* |
| 19 | Qualitative review on domains of quality of life important for patients, social care users, and informal carers to inform the development of the EQ-HWB (Mukuria et al., 2022) | United Kingdom, European countries,  Systematic review of qualitative studies,  Caregivers (aged ≥18 years) of mixed populations | - Active involvement in social activities - Meaningful relationships   This review synthesized qualitative evidence on quality of life from studies involving selected health conditions, informal carers, and social care users to inform the development of a new quality-of-life measurement tool. Social engagement was identified as a subtheme within the relationship’s domain. It refers to engaging in social activities and maintaining meaningful relationships with others. Unlike social support, which generally refers to receiving assistance from a broader social environment or network, social engagement emphasizes connection, inclusion, and emotional bonds. | Consequences:   - Caregiver health and psychosocial well-being (Quality of life)   *"Engaging in social activities and maintaining friendships were also seen as important for quality of life."* |
| 20 | Grandchild care and well-being: Gender differences in mental health effects of caregiving grandparents (Notter, 2022) | United States,  Quantitative study,  Caregivers (aged ≥50 years) of grandchildren | - Active involvement in social activities - Shared and supportive contexts   This study highlighted that social engagement was positively associated with grandparents’ mental health. Also, it acted as a protective factor for grandmothers by buffering the negative impact of skipped-generation caregiving on mental health. Social engagement is commonly assessed through indicators of social connectedness. It involves engaging in formal activities, including religious services, political involvement, and informal group gatherings, and it is distinct from providing childcare. | Antecedents:   - Caregivers’ gender   *"Taken together, I provide causal evidence that grandchild care and social engagement improve well-being outcomes, with only minimal differences by gender."*  Consequences:   - Caregiver health and psychosocial well-being **(**Mental health)   *"Care-providing grandparents who increase their social engagement with other family members, friends, and in volunteering activities show improvements to mental health."*  Empirical referents |
| 21 | An exploration into the experience of family caregivers for people living with dementia in a community dance class (Petts & Urmston, 2022) | United Kingdom,  Qualitative study,  Informal caregivers (mean age = 50.3 years) of PWD | - Active involvement in social activities - Shared and supportive contexts - Meaningful relationships   This qualitative study coded social engagement as one of the themes in the community dance experience for family caregivers. Participants described that the community dance activity brought them happiness and helped them feel connected to others. This supportive environment allowed them to talk openly about caring for their loved one, which helped reduce isolation and acknowledged their caregiving role. | Antecedents:   - Access to formal activities (Government financial support)   *"I don’t know how it’s funded I don’t know how long we can keep going but we’ll keep going until they say you can’t come any more."*  Consequences:   - Caregiver health and psychosocial well-being (Respite)   *"Participant 3 also admitted she was ‘looking to escape’ from her daily caregiving responsibilities and did so in dance classes."*   - Preserved identity (Caregiver)   *"Things are kind of less, and so you do dance like this, it kind of expands, says, “no you’re not just that [a carer] actually there is a lot more, it empowers you."*   - Caregiver health and psychosocial well-being (social isolation)   *"The joint participation in social dance activity, and the community it empowers, appears to help caregivers in this study to feel a reduced sense of isolation and increased validation in their caregiving experiences."*   - Preserved identity (Care recipient)   *"Dancing not only allowed the participants to reconnect but also to reminisce, enabling a sense of coherence with past memories made together."* |
| 22 | When missing out matters: Associations between social activity restriction and caregiver burden  (Rosenberg et al., 2026) | United States,  Quantitative study,  American informal caregivers (mean age = 62.5 years) of older adults | - Active involvement in social activities - Meaningful relationships   This study examined the association between social activity restriction and caregiver burden among 1,619 unpaid caregivers of older adults. The authors hypothesized that the association would be stronger when caregivers were unable to engage in activities they considered personally meaningful. Social engagement was operationalized as participation in social activities, specifically visiting friends or family, attending religious services, participating in clubs or other organized activities, and going out for enjoyment. Caregivers also rated the importance of each activity as very important, somewhat important, or not so important. Greater restriction from these activities was associated with higher caregiver burden. Caregiver burden increased further as the number of missed activities increased. However, perceived importance was associated with greater burden only for missed religious activities. The authors suggested that social engagement may provide caregivers with a sense of belonging, identity, and fulfillment. | Antecedents:   - Constriction of social life   *"Caregivers are at particular risk for social activity restriction due to the time demands of their care responsibilities."*  Consequences:   - Caregiver health and psychosocial well-being (Caregiving burden)   *"Based on prior conceptual frameworks and study results, we hypothesize that social restriction contributes to caregiver burden."*   - Caregiver health and psychosocial well-being (Stress)   *"Social engagement can improve emotional regulation and reduce stress regardless of perceived importance."*   - Caregiver health and psychosocial well-being **(**Mental health)   *"Social engagement can improve emotional regulation and reduce stress regardless of perceived importance."*  Empirical referents |
| 23 | Social support and self-rated health of African American women informal caregivers: Urban and rural differences (Rozario & Simpson, 2018) | United States,  Quantitative study,  African American informal caregivers (urban mean age = 55.3 years; rural mean age = 52.1 years) of older adults | - Active involvement in social activities - Shared and supportive contexts - Meaningful relationships   This study investigated the influence of geographic region on social support and its association with self-rated health among African American women informal caregivers of older adults. Social engagement, conceptually defined as participation in one’s social network, was one of three dimensions of support, alongside structural (social network size) and functional (relationship satisfaction) aspects. Operationally, social engagement was measured by the frequency of interactions with non–co-residing relatives or members of one’s broader social network. | Antecedents:   - Geographical region   *"significantly more urban caregivers reported receiving assistance from others and having more regular contact with their family than rural caregivers."*  Consequences:   - Caregiver health and psychosocial well-being   *"Both urban and rural caregivers reporting positive perceptions of one’s family functioning had significantly higher odds of reporting better self-rated health."*  Empirical referents |
| 24 | Fit for caring: factors associated with informal care provision by older caregivers with and without multimorbidity (Schmidt et al., 2016) | European countries,  Quantitative study,  Informal older caregivers (≥60 years) providing care to adults or grandchildren | - Active involvement in social activities   This study investigated the factors influencing care provision among older European informal caregivers, both with and without multimorbidity. Social engagement in this study was conceptually defined as active involvement in caring activities and operationally defined as the provision of extra-residential informal care to vulnerable family members, friends, or neighbors, as well as non-custodial grandparenting. Additionally, other activities such as religious involvement, leisure, and volunteering were also considered forms of social engagement. | Antecedents:   - Caregivers’ baseline health   *"The positive association with religious activities (especially for those without multimorbidity) underlines the strong altruistic character of informal care provision."*   - Caregivers’ marital status   *"Being married increases the probability of providing grandchild care in both groups."*   - Caregivers’ gender   *"We ﬁnd that women are (almost 5 percentage points) more likely to be involved than men for grandchild care."*   - Caregivers’ educational status   *"Higher income and education level are strongly associated with the provision of care in the non-multimorbidity group."*   - Household size   *" Larger household size is positively associated with grandchild care among those with multimorbidity."*   - Caregivers’ employment status   *"Labor market participation is negatively associated with grandparenting among those in better health."*   - Caregivers’ income   *"Higher income and education level are strongly associated with the provision of care in the non-multimorbidity group."*  Empirical referents |
| 25 | Remini-sing: A feasibility study of therapeutic group singing to support relationship quality and wellbeing for community-dwelling people living with dementia and their family caregivers (Tamplin et al., 2018) | Australia,  Mixed-methods study,  Informal caregivers (mean age = 73.9 years) of PWD | - Active involvement in social activities - Shared and supportive contexts - Meaningful relationships   This feasibility study evaluated the acceptability of using therapeutic group singing to support the relationship quality and well-being of caregiving dyads of people with dementia. Engaging in group singing was described as a form of social engagement, in which individuals shared common interests and dementia caregiving experiences in a nonjudgmental environment. Participants reported feelings of connection and enjoyment, and this relational quality remained high even at the end of the 20-week intervention. | Antecedents:   - Engagement activity group size   *"It’s sort of the size of the group as well. Um, you can get around everybody. Whereas if it was any bigger, I think that might cause some problems."* |
| 26 | Remini-sing RCT: Therapeutic choir participation for community-dwelling people with dementia and their primary caregivers (Tamplin et al., 2024) | Australia,  Quantitative study,  Informal caregivers (control mean age = 70.0; intervention mean age = 64.0) of PWD | - Active involvement in social activities - Shared and supportive contexts - Meaningful relationships   This randomized controlled trial enrolled 16 caregiving dyads of PWD in the intervention group and 18 in the waitlist control group to examine the effects of a therapeutic choir intervention on their relationship quality and well-being. The authors considered social engagement to be active, joint participation in this social and musical intervention, which provided enjoyment and shared experiences that fostered mutual understanding within caregiving dyads. However, no significant results were found, likely due to insufficient power resulting from recruitment challenges. | N/A |
| 27 | Social Consequences of advanced cancer in patients and their informal caregivers: a qualitative study (van Roij et al., 2019) | Netherlands,  Qualitative study,  Informal caregivers (mean age = 58.0 years) of people with advanced cancer | - Active involvement in social activities   This qualitative study explored the social Consequences of advanced cancer on patients and their informal caregivers. Themes were developed through thematic analysis and included social engagement, social identity, and social networks. Caregiving demands restricted their social engagement, such as participating in leisure activities or maintaining paid work. Some caregivers also reported feelings of shame or guilt when engaging in other activities. However, they continued to strive for a sense of normality in their daily lives. | Antecedents:   - Intrapsychic strain   *"At that time I made a conscious decision to continue playing golf; it is something that enables me to clear my head, and that is extremely important to me. But it is difficult, because you are away for four or five hours at a time which is often rather too long for [PATIENT]."*   - Constriction of social life/ Intrapsychic strain   *"However, most informal caregivers spend less time on social activities for multiple reasons, ... difficult to leave the patient due to feelings of selfishness, shame, worries, or being judged by others."* |
| 28 | Invisible Walls: A Concept Analysis of Social Isolation in Family Caregivers  (Wang et al., 2026) | Thailand, concept analysis, Family caregivers across caregiving contexts | - Active involvement in social activities   This concept analysis aimed to define and clarify social isolation in family caregivers and identify its attributes, antecedents, Consequences, and empirical referents. Reduced social engagement was identified as one of five defining attributes of social isolation and was characterized as a behavioral or functional limitation. Caregivers may actively reduce participation in social or leisure activities, limit contact with family, friends, and social groups, and have difficulty sustaining their usual social roles and routines. | N/A |
| 29 | A psychosocial intervention for carers of individuals diagnosed with dementia in social isolation (Wei et al., 2023) | Australia,  Quantitative study,  Informal caregivers (mean age = 64.9 years) of PWD | - Shared and supportive contexts   This pre–post intervention study was designed to evaluate the effectiveness of an online psychoeducation toolkit for dementia caregivers during the COVID-19 pandemic. Social engagement was operationalized as social network size, defined as the number of people with whom the caregiver interacted in the past two weeks. The results showed that the psychosocial intervention increased participants’ social network size, which may help reduce social isolation among dementia caregivers. | Empirical referents |
| 30 | Grandparenting and cognitive functioning in China (Xu, 2022) | China,  Quantitative study,  Caregivers (aged 50-75 years) of grandchildren | - Active involvement in social activities - Shared and supportive contexts   This longitudinal study used secondary data to analyze the influence of grandparenting on cognitive function among people who had cared for their grandchildren over the past year. Grandparenting was considered a form of social engagement for grandparents, as it could support them in actively engaging with others in later life. Such engagement includes sharing stories, enjoying leisure activities, and teaching important life values to their grandchildren. | Antecedents:   - Caregivers’ gender/ Geographic region   *"Taken together, these findings suggest that Chinese grandparents enjoy cognitive benefits from providing casual or intensive care to grandchildren, but these benefits vary substantially by gender and rural–urban status."*  Consequences:   - Caregiver health and psychosocial well-being (Cognitive health)   *"Providing part-time care to grandchildren was associated with higher scores on episodic memory for both rural and urban grandfathers."*  Empirical referents |
| 31 | Beyond close ties: how social engagement shapes care-giver  burden in later life  (Zhong et al., 2026) | United States,  Quantitative study,  Caregivers (mean age = 70.7 years) of mixed populations | - Active involvement in social activities - Shared and supportive contexts   This secondary data analysis examined associations between two dimensions of social connectivity and caregiver burden among older adult caregivers. Social connectedness was characterized by features of an individual’s core social network, while social engagement was defined as the enactment of social ties through real-life activities and participation in social activities. Social engagement was further conceptualized as opportunities for interactions with casual acquaintances and was operationalized using the number of friends and the frequency of volunteering, attending organized group meetings, and attending religious services. Findings showed that higher levels of all four social engagement indicators were associated with lower odds of caregiver burden. More frequent and more diverse social engagement across activity types was also associated with lower caregiver burden. In contrast, a larger core social network of strong ties was associated with higher odds of caregiver burden. | Consequences:   - Caregiver health and psychosocial well-being (Caregiving burden)   *"Regarding broader social engagement, all types decreased caregivers’ odds of experiencing caregiver burden."*  Empirical referents |

**Table S3**

*Studies reporting empirical referents for family caregiver social engagement with corresponding example quotations*

| Author, year | Items | Response Format/ Scoring System | Operationalized defining attributes (Operational Concept) | | | Others | | | | |
| --- | --- | --- | --- | --- | --- | --- | --- | --- | --- | --- |
|  |  |  | **Active involvement in social activities**  (Number of activity types/  Frequency of contact) | **Shared and supportive contexts**  (Activity involving shared contexts/Social network size) | **Meaningful relationships**  (Satisfaction with/ Importance of activities) | | | | |  |
| Babenko et al., 2026 | 5 | 4–7 frequency options/  Yes | Yes,  "In the past 12 months, how often did you attended religious services, attended organized group meetings, did volunteer work, got together socially with friends or relatives, and visited neighbors" (*Frequency*) | No | No | | | No | | |
| Burn & Szoeke, 2015 | 2 | Binary, Numeric entry/  No | Yes  "Those who answered “yes” were then asked about the frequency of care." (*Frequency*) | No | No | | | No | | |
| Gorenko et al., 2024 | 6 | 3-point scale/ Yes | Yes  "The study assessed the frequency of social activities over the past six months (e.g., interest, dining out, social activities, visiting family or friends)." (*Frequency*) | Yes  Include "visiting family or friends." (*Activity contex*t) | No | | | No | | |
| Gudenkauf et al., 2024 | 6 | 5-point scale/  Yes | No | Yes  "The question includes assessing the trouble doing leisure activities, family activities, usual work, and activities with friends." (*Activity contex*t) | No | | Yes  "This 6-item scale uses a 5-point scale, with higher scores indicating greater social engagement." | | | |
| Jennings et al., 2021 | 2 | 7-point scale, Numeric entry/  Yes | Yes  "Social interaction was measured by the average number of days per month grandparents interacted with up to seven named contacts over the past six months." (*Frequency*) | No | No | | | No | | |
| Kelley et al., 2017 | 5 | Binary /  Yes | Yes  "Social engagement was measured by the number of social activity types that caregivers participated in during the past month." (*Types*) | Yes  "Engaging in religious services, volunteer work, visits with friends or family, club or group meetings, and recreational outings." (*Activity contex*t) | No | | | No | | |
| Liang et al., 2020 | 5 | Binary /  Yes | Yes  "Social engagement was measured by the number of social activity types that caregivers participated in during the past month." (*Types*) | Yes  "Engaging in religious services, volunteer work, visits with friends or family, club or group meetings, and recreational outings." (*Activity contex*t) | No | | | No | | |
| Litzelman et al., 2020 | 7 (*Types*)  1 (*Satisfaction*) | Binary /  3-point scale  Yes | Yes  "Social engagement was measured based on caregivers’ responses regarding their regular use of seven types of social resources." (*Types*) | Yes  "Including formal resources such as counseling or psychotherapy, support groups, health-related groups, and online support, and informal resources like social clubs, faith-based groups, and others." (*Activity contex*t) | Yes  "Satisfaction with the caregiving help received was measured by one item using a 3-point scale (low-medium satisfaction, quite satisfied, or very satisfied)."  (Satisfaction with activities) | | | No | | |
| Liu et al., 2021 | 2 (*Social* n*etwork size*)  1 (*Frequency*)  5 (*Satisfaction*) | Numeric entry  6-point scale  3-point scale/  Yes | Yes  "Monthly social contact was assessed by the third question: “How many of these friends or relatives do you see at least once a month?" (*Frequency*) | Yes  "The first two questions from the Revised Lubben Social Network Scale (LSNS): “(1) How many close friends do you have?” and “(2) How many relatives do you have that you feel close to?" (*Activity contex*t/*Social network size*) | Yes  "Additionally, participants rated their satisfaction with time spent in five social activities over the past month only in the second interview." (Satisfaction with activities) | | | No | | |
| Liu et al., 2023 | 2 (*Social* *network size*)  1 (*Frequency*) | Numeric entry  6-point scale | Yes  "Monthly social contact was assessed by the third question: “How many of these friends or relatives do you see at least once a month?" (*Frequency*) | Yes  "The first two questions from the Revised Lubben Social Network Scale (LSNS): “(1) How many close friends do you have?” and “(2) How many relatives do you have that you feel close to?" (*Activity contex*t/*Social network size*) | No | | | No | | |
| Notter, 2022 | 4 | Binary/  Yes | Yes  "Four relevant items from the Health and Retirement Study database: being married or in a partnership, having weekly contact with at least one parent or parent-in-law, having weekly contact with friends, and volunteering at least one hour per week. Each item was scored as “yes” (1) or “no” (2)." (*Types*) | Yes  "Having weekly contact with at least one parent or parent-in-law, having weekly contact with friends, and volunteering at least one hour per week." (*Activity contex*t) | No | | | No | | |
| Rosenberg et al., 2026 | 4 (*Types*)  4 (*Importance*) | Binary  3-point scale/  Yes | Yes  "Social engagement was measured by the number of social activity types that caregivers participated in during the past month." (*Types*) | No | Yes No  "Respondents were asked how important that activity was to them: “very important,” “somewhat important,” or “not so important.”" (Importance of activities) | | | | | |
| Rozario & Simpson, 2018 | 2 | 6-point scale/  Yes | Yes  "How often do you [caregiver] see, write to, or talk on the telephone with family or relatives who do not live with you?" and (2) "How often do you [caregiver] attend religious services?" (*Frequency*) | Yes  "How often do you [caregiver] see, write to, or talk on the telephone with family or relatives who do not live with you?" and (2) "How often do you [caregiver] attend religious services?" (*Activity contex*t) | No | | | No | | |
| Schmidt et al., 2016 | 2 | Binary/  No | Yes  "Asking whether the participant had, within the past 12 months, (1) provided extra-residential care to adults, such as assistance with personal care, household chores, or paperwork, and (2) provided grandchild care in the absence of the children’s parents." (*Types*) | No | No | | | No | | |
| Wei et al., 2023 | 1 | Numeric entry/  No | No | Yes  "Carers’ social network size was quantified as the number of people in their social circle that they saw or spoke to on a regular basis." (*Social network size*) | No | | | No | | |
| Xu, 2022 | 2 | Binary, Numeric entry/  No | No | No | No | | Yes  "Grandparenting was measured by asking whether participants provided care, and if so, how many weeks and hours per week they spent caregiving." | | | |
| Zhong et al., 2026 | 3 (*Frequency*)  3 (*Types*)  1 (*Social network size*) | 7-point scale,  6-point scale/  Yes | Yes  "In the past 12 months, how often did you do volunteer work, attend meetings of any organized group, attend religious services" (*Frequency* )  Breadth/diversity was derived from the frequency items by recoding each activity as weekly or more and summing the number of activity types (*Types*) | Yes  "About how many friends do you think you have?" (*Social network size*) | No | | No | | | |
| 17 Articles | 1–8 items |  | 7 (*Types*)  8 (*Frequency*) | 9 (*Activity contex*t)  4 (*Social network size*) | 2 (Satisfaction with activities)  1 (Importance of activities) | | | | 2 | |
